# Supplementary material for: A revised turtle assemblage from the Upper Cretaceous Menefee Formation (New Mexico, North America) with evolutionary and paleobiostratigraphic implications
Source: PeerJ. 2025 Apr 23;13:e19340. doi: 10.7717/peerj.19340 (PMC12032963; doi:10.7717/peerj.19340)
Supplement: Supplemental Information 1 — Asterisks (“ * ”) indicate approximate measurement. Measurements for USNM 8344 are from the original description of Gilmore (1916: 290). [file peerj-13-19340-s001.docx]

Supplementary Information

Table S1. Raw measurement data in millimeters (mm) for *Neurankylus baueri* specimens WSC 10612 from the Menefee Formation, and USNM 8344 (holotype) and SMP VP-2379 from the Hunter Wash Member of the Kirtland Formation (Sullivan, Jasinski & Lucas, 2013). Measurements of USNM 8344 are taken from Gilmore (1916). Asterisks (“*”) indicates measurement of partial element.

| **Measurement** | **WSC 10612** | **USNM 8344** | **SMP VP-2379** |
| --- | --- | --- | --- |
| Vertebral 1 scale |  |  |  |
| Length | 93.6 | 81.0 |  |
| Width | 135.3 | 133.0 |  |
| Vertebral 2 scale |  |  |  |
| Length | *80.3 | 106.0 |  |
| Width | 136.8 | 165.0 |  |
| Inframarginal 1 scale |  |  |  |
| Length | 66.0 |  |  |
| Width | 60.6 |  |  |
| Carapace thickness at middle of costal 1, left side | 19.0 |  |  |
| Anterior carapace sulcus width | 2.4 |  |  |
| Cervical scale |  |  |  |
| Length | 24.3 | 19.0 | 20.8 |
| Width | *21.6 | 14.0 | 13.2 |
| Anterior plastral lobe |  |  |  |
| Length | 73.0 | 111.0 | 119.8 |
| Width | 128.5 | 168.0 | 200.9 |
| Posterior plastral lobe |  |  |  |
| Length |  | 122.0 | *131.3 |
| Width | *75.0 | 185.0 | 209.2 |
| Plastral scale midline contacts |  |  |  |
| Gular | 38.9 | 40.0 | 46.3 |
| Humeral | 65.7 | 79.0 | 64.1 |
| Pectoral | 107.7 | 100.0 | 93.8 |
| Abdominal | 71.2 | 60.0 | 74.3 |
| Maximum carapace length | 584.0 (est.) | 560.0 | 569.7 |
